# Phylogeographic analysis reveals multiple origins of the desert shrub *Reaumuria songarica* in northern Xinjiang, involving homoploid and tetraploid hybrids

**DOI:** 10.1002/ece3.70199

**Published:** 2024-08-29

**Authors:** Xingke Fan, Xia Yan, Chaoju Qian, Ibrahim Awuku, Pengshu Zhao, Yuqiu Liao, Zhijun Li, Xinrong Li, Xiao‐Fei Ma

**Affiliations:** ^1^ Key Laboratory of Ecological Safety and Sustainable Development in Arid Lands Northwest Institute of Eco‐Environment and Resources, Chinese Academy of Sciences Lanzhou China; ^2^ Key Laboratory of Stress Physiology and Ecology in Cold and Arid Regions, Department of Ecology and Agriculture Research, Northwest Institute of Eco‐Environment and Resources, Chinese Academy of Sciences Lanzhou China; ^3^ Key Laboratory of Eco‐Hydrology of Inland River Basin, Northwest Institute of Eco‐Environment and Resources Chinese Academy of Sciences Lanzhou China; ^4^ College of Pastoral Agriculture Science and Technology Lanzhou University Lanzhou China; ^5^ University of Chinese Academy of Sciences Beijing China; ^6^ Key Laboratory of Protection and Utilization of Biological Resources in Tarim Basin Xinjiang Production and Construction Corps Alar China; ^7^ Shapotou Desert Research and Experiment Station Northwest Institute of Eco‐Environment and Resources, Chinese Academy of Sciences Lanzhou China

**Keywords:** climatic oscillation, homoploid hybrid speciation, Junggar Basin, multiple origins, plant diversity, *Reaumuria songarica*

## Abstract

Hybrid speciation plays an important role in species diversification. The establishment of reproductive isolation is crucial for hybrid speciation, and the identification of diverse types of hybrids, particularly homoploid hybrid species, contributes to a comprehensive understanding of this process. *Reaumuria songarica* is a constructive shrub widespread in arid Central Asia. Previous studies have inferred that the *R. songarica* populations in the Gurbantunggut Desert (GuD) originated from homoploid hybridizations between its eastern and western lineages and may have evolved into an incipient species. To further elucidate the genetic composition of different hybrid populations and to determine the species boundary of this hybrid lineage, we investigated the overall phylogeographic structure of *R. songarica* based on variation patterns of five cpDNA and one nrITS sequences across 32 populations. Phylogenetic analyses demonstrated that within the GuD lineage, the Wuerhe population evolved directly from ancestral lineages, whereas the others originated from hybridizations between the eastern and western lineages. PCoA and genetic barrier analysis supported the subdivision of the GuD lineage into the southern (GuD‐S) and northern (GuD‐N) groups. Populations in the GuD‐S group had a consistent genetic composition and the same ancestral female parent, indicating that they belonged to a homoploid hybrid lineage. However, the GuD‐N group experienced genetic admixture of the eastern and western lineages on nrITS and cpDNA, with some populations inferred to be allopolyploid based on ploidy data. Based on cpDNA haplotypes, BEAST analyses showed that the GuD‐S and GuD‐N groups originated after 0.5 Ma. Our results suggest that multiple expansions and contractions of GuD, driven by Quaternary climatic oscillations and the Kunlun‐Yellow River tectonic movement, are important causes of the complex origins of *R. songarica* populations in northern Xinjiang. This study highlights the complex origins of the Junggar Basin flora and the underappreciated role of hybridization in increasing its species diversity.

## Graphical abstract

The *Reaumuria songarica* in northern Xinjiang contains an ancient population, homoploid hybrid populations, and tetraploid hybrids. These hybrid populations originated from different hybridization events between the eastern and western lineages and can be divided into the GuD‐S (incipient homoploid hybrid species) and GuD‐N genetic groups. The multiple expansions and contractions of the Gurbantunggut Desert are important drivers of the complex origins of *R. songarica* in northern Xinjiang.

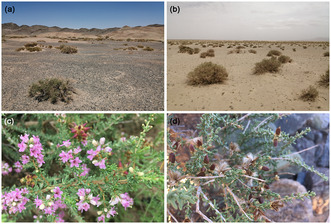

## 1 INTRODUCTION

Hybridization between or within species is common in nature and has been recognized as an important driver of evolution and speciation (Abbott et al., 2013; Barton, 2001; Mallet, 2007). In the plant kingdom, the proportion of species involved in hybridization and introgression is estimated to be as high as 25% (Mallet, 2005). Numerous empirical studies have demonstrated that hybridization can lead to a variety of evolutionary outcomes, including adaptive introgression, the formation or extinction of hybrid zones, the reversal of speciation, and even the formation of new species (hybrid speciation) (Jiang et al., 2016; Kearns et al., 2018; Wang et al., 2021; Wong et al., 2022; Yu et al., 2023). Although only fertile hybrids with high fitness and reproductive isolation (RI) from their parental species have the potential to evolve into independent species, numerous hybrid species from different families have been identified (Abbott et al., 2013; Mallet, 2007; Wang et al., 2021). Therefore, it is widely acknowledged that hybrid speciation plays a significant role in increasing species diversity (Soltis & Soltis, 2009; Wu et al., 2022). However, the frequency and process of hybrid speciation will be strongly affected as current climate change and human activities continue to break down ecological or geographic barriers between isolated species by moving them (Vallejo‐Marin & Hiscock, 2016).

Hybrid speciation includes two main types: homoploid hybrid speciation (HHS) and allopolyploid speciation. In HHS, the ploidy level of the hybrids is identical to that of their parental species, whereas in allopolyploid speciation, the hybrids have double the number of chromosomes (Mallet, 2007). In plants, HHS is less prevalent than allopolyploid speciation (Barker et al., 2016; Mallet, 2007; Schumer et al., 2014). Even if viable and fertile homoploid hybrids are formed, they face significant difficulties in rapidly establishing RI from the parental species due to the lack of a polyploidy barrier (Mallet, 2007). As a result, these hybrids are more likely to become extinct through successive backcrosses with the parental species (Buerkle et al., 2000). Establishing RI between hybrids and their parental species is therefore both challenging and crucial for the success of HHS. However, the evolution of RI in HHS remains controversial and the genetic mechanisms involved are far from clear. Most studies suggest that RI driven by ecological divergence plays a major role in promoting HHS (Gross & Rieseberg, 2005; Mallet, 2007; Zhao et al., 2014). However, several recent studies using population genomic data indicate that the new combination of parental premating and/or postmating isolating genes and barriers, driven by hybridization, is an important mechanism for establishing RI in HHS (Rosser et al., 2024; Sun et al., 2020; Wang et al., 2021; Wu et al., 2023). Unfortunately, uncovering common mechanisms for the establishment of RI in HHS remains challenging due to the limited number and diversity of well‐documented homoploid hybrid species (Long & Rieseberg, 2024; Rieseberg et al., 2003; Zhao et al., 2014). To date, only the premating isolation genes between *Ostryopsis intermedia* and its parental species have been experimentally confirmed (Wang et al., 2021). Thus, identifying more diverse types of hybrid species and hybrid zones is essential to better understand the effects of hybridization on RI and adaptive transitions, and to explore the genetic basis of different evolutionary stages in HHS.

**FIGURE 1:**
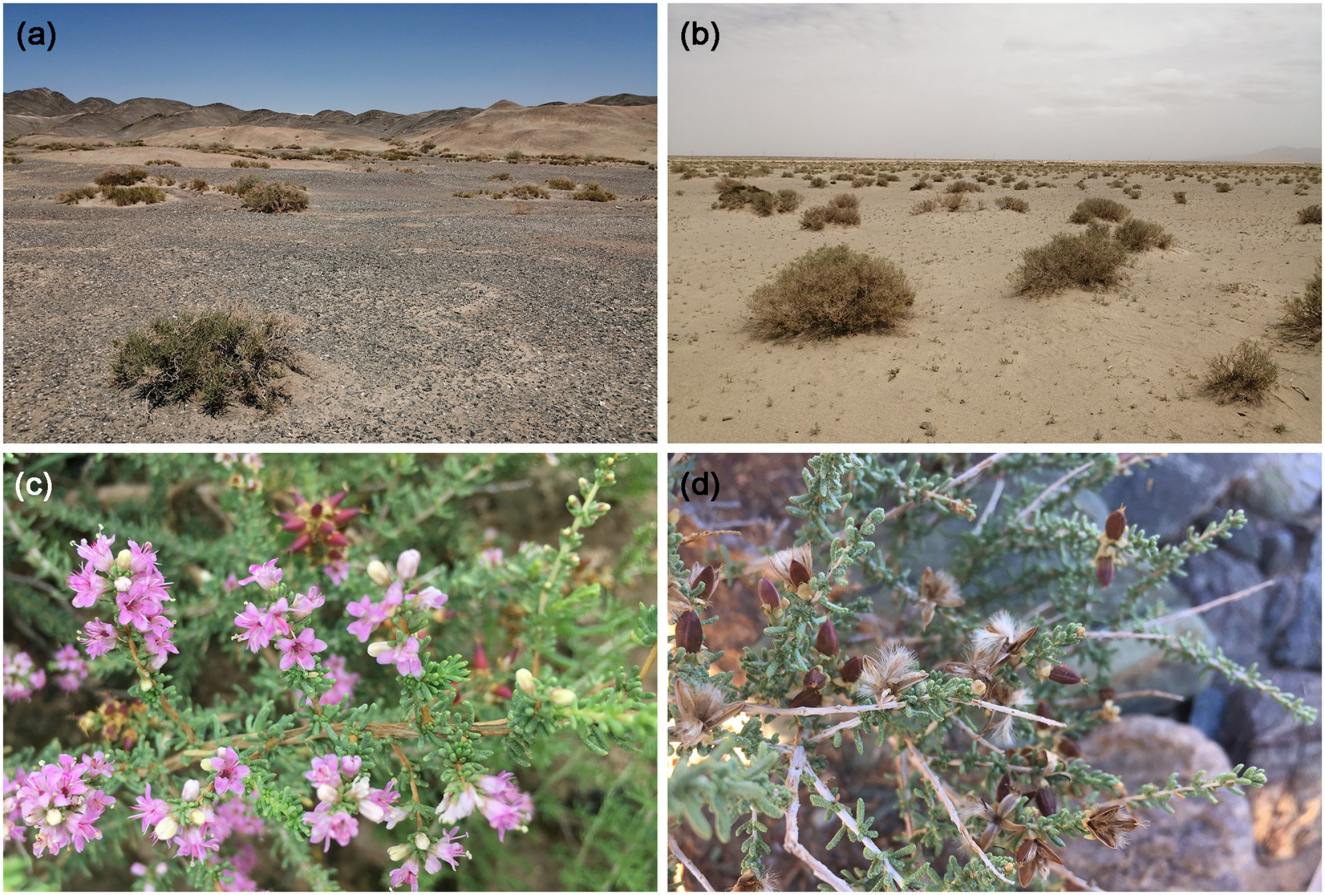
Different habitats and organs of *Reaumuria songarica*. (a) Gravel desert; (b) sandy land without dunes; (c) flowers and buds; (d) the seeds are densely covered with long trichomes, allowing for long‐distance dispersal by the wind (Zeng et al., 2002).

*Reaumuria songarica* (Figure 1), a member of the Tamaricaceae family, is a highly xerophytic shrub that is widespread in the temperate deserts of arid Central Asia (ACA), except in sand dunes (Liu et al., 1982; Shi et al., 2013). Its reproductive strategies include seed germination, root splitting, and adventitious root propagation. In arid conditions, populations primarily rely on vegetative reproduction (Zeng et al., 2002). As a common constructive and dominant species, *R. songarica* is essential for maintaining the stability and biodiversity of the fragile desert ecosystems (Ma et al., 2005). Previous phylogeographic studies, based on maternally inherited chloroplast DNA (cpDNA) markers and biparentally inherited nuclear ribosomal internal transcribed spacer (nrITS) sequences (Liu et al., 2009), have shown that this species diverged into the eastern and western genetic/geographic lineages, driven by the uplift of the Qinghai‐Xizang Plateau (QXP) and the development of the East Asian monsoon system (EAMS) (Li et al., 2012; Yin et al., 2015). The variation patterns of multiple nuclear loci further suggest that the GuD genetic lineage (also referred to as the northern lineage), distributed in the Gurbantunggut Desert in northern Xinjiang, originated from homoploid hybridizations between the eastern and western lineages (Shi et al., 2020). Despite experiencing continuous gene flow from its parental lineages, the GuD lineage has a flowering time between the non‐overlapping flowering times of the parents, and its genetic composition, ecological niche, and leaf size are significantly different from those of its parents (Fan et al., 2020; Shi et al., 2020). According to the integrative species concept (Liu, 2016), the GuD lineage and its parental lineages could be incipient species (Fan et al., 2021; Shi et al., 2020). Unlike most homoploid hybrid species, which show strong RI from their parental species (Wang et al., 2021; Zhao et al., 2014), the emerging GuD lineage offers a valuable opportunity to gain deeper insights into the processes and mechanisms underlying the early stages of HHS, such as the evolution of RI.

The Gurbantunggut Desert in the Junggar Basin, surrounded by mountains on all sides, is the highest‐latitude desert in China. The environmental factors across the different regions of this desert exhibit significant variations, such as precipitation, temperature, and wind direction (Jiang et al., 2015; Liu, 2020). Based on field observations, we found that *R. songarica* is distributed around the edges of the Gurbantunggut Desert. However, the populations of the GuD lineage analyzed in previous studies were located only at the southeastern edge of this desert (Li et al., 2008; Shi et al., 2020; Yin et al., 2015). Furthermore, the genetic compositions of these studied populations vary considerably. For example, the haplotypes of cpDNA and nrITS in the Shawan population are derived from the eastern and western lineages, respectively; conversely, the Huoshaoshan population is dominated by the cpDNA haplotypes of the western lineage and the nuclear genetic components of the eastern lineage (Shi et al., 2020; Yin et al., 2015). Therefore, we hypothesized that there is a high degree of genetic differentiation among the hybrid populations of *R. songarica* across different regions of the Gurbantunggut Desert. However, the overall population structure of the GuD lineage remains unclear, which limits the accurate and in‐depth study of the speciation mechanism of this hybrid lineage.

In most seed plants, cpDNA is uniparentally inherited (Birky, 1995; Wicke et al., 2011), so its patterns of genetic variation among different populations can provide valuable information on the origin and colonization routes of the species under investigation (Qian et al., 2016; Wang et al., 2011). However, phylogenetic analyses based on cpDNA markers alone can hardly reveal introgression or hybridization events that may have occurred during the speciation. Combined analysis of the nuclear and chloroplast DNA markers can overcome this limitation and has become an essential method in phylogeographic studies (Morris & Shaw, 2018). Here, we used both cpDNA and nrITS markers to fully elucidate the phylogeographic structure of the GuD lineage and its parental lineages in order to address the above hypotheses. Specifically, we aimed to (a) investigate the population structure of the GuD lineage and identify the ancestral parents of the various hybrid populations; (b) determine the exact distribution range of the homoploid hybrid populations; and (c) explore the factors that have caused and maintained higher genetic divergence within this hybrid lineage.

## 2 MATERIALS AND METHODS

### 2.1 Sample collection and data acquisition

A total of 243 individuals from 32 populations of *R. songarica* were selected for subsequent phylogeographic analyses (Table S1). Based on previous studies (Shi et al., 2020; Yin et al., 2015), these populations are believed to represent distinct genetic groups and lineages. The western lineage includes the TaD group, the GuD lineage consists of the GuD group, and the eastern lineage contains the BJTD and QBKG groups. Furthermore, the locations of the populations studied covered the entire range of *R. songarica* in northwestern China. Of these, 13 populations were newly sampled and sequenced in this study, including the CDY, ELHT, and MG populations, as well as 10 populations located in northern Xinjiang. In each new population, fresh leaves were collected from three to nine individuals spaced at least 30 meters apart and then dried using silica gel. These voucher specimens are deposited in the Key Laboratory of Stress Physiology and Ecology in Cold and Arid Regions, Northwest Institute of Eco‐Environment and Resources, Chinese Academy of Sciences.

Additionally, we obtained the published sequences of eight individuals from each remaining *R. songarica* population and three individuals from each outgroup (*Tamarix amplexicaulis* and *Myricaria pulcherrima*) from a previous study (Yin et al., 2015). This downloaded dataset included the sequences of five cpDNA fragments (*pet*B‐*pet*D, *trn*S2‐*trn*G2, *atp*H‐*atp*I, *ndh*A, and *pet*N‐*psb*M) and one nrITS fragment (ITS1‐ITS4), which have polymorphic sites among *R. songarica* populations (Yin et al., 2015).

### 2.2 DNA extraction, amplification, and sequencing

Total DNA of *R. songarica* leaves was extracted and purified using a Plant Genomic DNA Kit [DP305, TIANGEN BIOTECH (Beijing) Co., Ltd, China]. All six DNA fragments mentioned above were amplified and sequenced in the 91 newly collected individuals. Detailed information on their primers is provided in Table S2. The Polymerase Chain Reaction (PCR) amplification procedures followed the protocol presented by Yin et al. (2015), and only the annealing temperature of *pet*B‐*pet*D, *trn*S2‐*trn*G2, and *atp*H‐*atp*I were modified in this study. Subsequently, the PCR products were sequenced using corresponding forward and reverse primers on an ABI 3730xl DNA Analyzer at Sangon Biotech (Shanghai, China).

Using Chromas version 2.6.5 (http://technelysium.com.au/) to view the chromatogram file for each sequence, we carefully proofread these newly acquired sequences and removed low‐quality DNA bases at their start and end positions. Meanwhile, the heterozygous sites in the nrITS sequences were carefully identified following the method described by Aguilar and Feliner (2003). All processed and downloaded sequences from each fragment were aligned using Clustal W implemented in BioEdit version 7.2.5 (Hall, 1999), and the alignments were then manually improved. Singletons were confirmed by resequencing to exclude sequencing errors. The five cpDNA fragments were then assembled into a supergene in the order described above using DnaSP version 5.10.01 (Librado & Rozas, 2009). Different sequences of this cpDNA supergene were identified as haplotypes (hereafter referred to as chlorotypes). The nrITS sequence of each individual was divided into two haplotypes (hereafter referred to as ribotypes) using the PHASE program integrated into the DnaSP software.

### 2.3 Analyses of genetic diversity and phylogeny

The classic population genetic parameters (Table 1) of the cpDNA and nrITS sequences were calculated for each population and each lineage using DnaSP version 5.10.01. To analyze the phylogenetic relationships among chlorotypes and among ribotypes, their respective genealogical topologies were constructed using the median‐joining algorithm in NETWORK version 5.0.1.1 (Bandelt et al., 1999).

**TABLE 1:** Genetic information of cpDNA haplotypes in each *Reaumuria songarica* population.

Lineage	Group	Population code	N	S	h	Hd	Π	θw	cpDNA haplotypes (individuals)
GuD	GuD		81	16	7	0.653	0.0010	0.0009	
		HSS	8	0	1	0.000	0.0000	0.0000	C1(8)
		WCC	6	9	2	0.533	0.0013	0.0011	C1(4) C2(2)
		QKET	7	0	1	0.000	0.0000	0.0000	C2(7)
		DST	8	4	2	0.429	0.0005	0.0004	C2(6) C3(2)
		BEJ	7	9	3	0.524	0.0009	0.0010	C2(5) C3(1) C8(1)
		FK	9	2	3	0.417	0.0001	0.0002	C3(7) C4(1) C5(1)
		ML	8	0	1	0.000	0.0000	0.0000	C3(8)
		JH	8	0	1	0.000	0.0000	0.0000	C3(8)
		KT	8	0	1	0.000	0.0000	0.0000	C3(7) C6(1)
		SW	8	0	1	0.000	0.0000	0.0000	C3(8)
		WEH	4	0	1	0.000	0.0000	0.0000	C7(4)
Western	TaD		55	11	8	0.612	0.0003	0.0007	
		CDY	7	0	1	0.000	0.0000	0.0000	C9(7)
		BC	8	1	2	0.536	0.0001	0.0001	C16(5) C17(3)
		HT	8	3	3	0.464	0.0002	0.0003	C17(6) C24(1) C25(1)
		KS	8	5	2	0.250	0.0003	0.0005	C17(6) C29(1) C30(1)
		LP	8	1	2	0.250	0.0001	0.0001	C17(7) C32(1)
		MF	8	0	1	0.000	0.0000	0.0000	C33(8)
		KEKZ	8	1	2	0.536	0.0001	0.0001	C17(3) C27(5)
Eastern			107	25	22	0.890	0.0010	0.0013	
	BJTD		67	24	20	0.857	0.0008	0.0014	
		LHT	8	2	2	0.250	0.0001	0.0002	C10(7) C31(1)
		MQ	8	3	4	0.786	0.0004	0.0003	C10(3) C28(1) C35(3) C36(1)
		ZY	8	2	3	0.607	0.0003	0.0002	C10(5) C28(1) C35(2)
		MG	3	1	2	0.667	0.0002	0.0002	C11(1) C12(2)
		JYH	8	4	4	0.750	0.0006	0.0004	C2(2) C3(4) C10(1) C26(1)
		WD	8	4	4	0.750	0.0005	0.0004	C2(4) C10(1) C13(2) C37(1)
		HSW	8	11	6	0.929	0.0014	0.0012	C18(1) C19(2) C20(2) C21(1) C22(1) C23(1)
		ELHT	8	4	3	0.679	0.0005	0.0004	C2(4) C3(1) C10(3)
		ALS	8	6	5	0.893	0.0007	0.0006	C2(1) C10(2) C13(2) C14(2) C15(1)
			40	10	7	0.800	0.0010	0.0006	
	QBKG	KLKH
		8	5	2	0.536	0.0007	0.0005	C17(5) C28(3)
		SSG	8	0	1	0.000	0.0000	0.0000	C17(8)
		YQ	8	8	4	0.750	0.0010	0.0008	C2(4) C17(2) C26(1) C28(1)
		MHG	8	7	3	0.714	0.0011	0.0007	C10(2) C28(2) C34(4)
JQ	8	1	2	0.250	0.0001	0.0001	C10(1) C13(7)
Total			243	40	34	0.887	0.0011	0.0018	

*Note*: The cpDNA haplotypes include sites with gaps. However, sequence gaps were not considered in the analysis of h, Hd, π, and θw.

Abbreviations: h, number of haplotypes; Hd, estimates of haplotype diversity; N, number of samples; S, number of polymorphic (segregating) sites; θw, Theta (per site) from S; π, nucleotide diversity within populations.

Moreover, we used Bayesian inference (BI) and maximum likelihood methods to reconstruct the phylogenetic tree of chlorotypes of *R. songarica*. In these analyses, the cpDNA concatenated sequences of *M. pulcherrima* and *T. amplexicaulis* were used as outgroups. The GTR + G model was confirmed as the best‐fit nucleotide substitution model for this cpDNA dataset using jModelTest version 2.1.4 (Darriba et al., 2012). The BI tree was constructed using MrBayes version 3.2.6 (Ronquist et al., 2012) with default settings, except that the length of the Markov Chain Monte Carlo (MCMC) was set to 5 million generations, and trees were sampled every 1000 generations. At the end of the run, the first 25% of the sampled trees were discarded as burn‐in, and the remaining trees were used to generate a consensus tree and calculate the posterior probabilities of its branches. PhyML version 3.1 (Guindon & Gascuel, 2003) was used to reconstruct the maximum likelihood tree. Tree topologies were searched from five random starting trees using the Best of NNI and SPR approaches. The support index of each branch of the maximum likelihood tree was estimated by 1000 bootstrap replicates. All phylogenetic trees were visualized and modified using FigTree version 1.4.3 (http://tree.bio.ed.ac.uk/software/figtree/).

### 2.4 Population structure analyses

The *G*_ST_ and *N*_ST_ parameters are two measures of the degree of genetic differentiation among populations (Pons & Petit, 1995, 1996). The *G*_ST_ parameter focuses only on haplotype frequencies, whereas *N*_ST_ considers both haplotype frequencies and similarities between haplotypes. When *N*_ST_ is significantly greater than *G*_ST_, it indicates that haplotypes with closer relationships tend to be distributed in adjacent areas; in other words, there is a distinct phylogeographic structure among populations (Pons & Petit, 1996). To determine whether the phylogeographic structure within the GuD lineage was significant, we calculated *G*_ST_, *N*_ST_, and population gene diversity (*H*_S_ and *H*_T_) for each genetic group based on the cpDNA and nrITS datasets, respectively, using PermutCpSSR version 1.2.1 with 1000 permutations (Pons & Petit, 1996). Additionally, different sources of genetic variation within *R. songarica*, as well as its fixation indices (*F*_CT_, *F*_SC_, and *F*_ST_), were estimated using analyses of molecular variance (AMOVA) provided by Arlequin version 3.5.1.2 (Excoffier & Lischer, 2010). The significance of these fixation indices was tested through 1000 permutations. Pairwise differentiation (*F*_ST_) between genetic groups was also computed using Arlequin with default settings.

To further examine the number of genetic groups (*K*) within the GuD lineage, spatial analyses of molecular variance (SAMOVA) were performed on the cpDNA datasets of the GuD lineage and all *R. songarica* populations separately using SAMOVA version 2.0 (Dupanloup et al., 2002). These analyses took into account both the cpDNA data and geographic locations, defining the adjacent populations with similar genetic components as a group. We tested *K* values from 2 to 10. For each test, the initial configuration was set to 100 with 10,000 iterations. Finally, the *K* value with the maximum *F*_CT_ was taken as the optimal grouping option.

In addition, principal coordinate analyses (PCoA) were conducted on the cpDNA and nrITS datasets using GenAlex version 6.5 (Peakall & Smouse, 2012) to detect the population structure of *R. songarica*. As the GenAlex program requires a minimum of five sequences per population, the nrITS data for the QKET and MG populations and the cpDNA data for the MG and WEH populations were excluded from these analyses.

### 2.5 Molecular dating

To estimate the divergence times among genetic lineages, Bayesian analyses were performed on chlorotype sequences using BEAST version 1.8.0 (Drummond et al., 2012). The input file was generated using BEAUti. Additionally, the GTR + G nucleotide substitution model, the lognormal relaxed molecular clock, and the “Coalescent: constant size” model of the tree prior were set for all simulations. Unfortunately, there are still no published pollen or fossil records of *Reaumuria* that could be used as a minimum age calibration for molecular dating. Recently, Yao et al. (2019) time‐corrected the molecular phylogeny of all families of Caryophyllales by using plastomes of 141 species and 10 related fossil records; the results from the BEAST analysis showed that the crown age of Tamaricaceae (including three genera, *Reaumuria*, *Tamarix*, and *Myricaria*) was ca. 43.66 million years ago (Ma) [95% highest posterior densities (HPD), 23.68–64.12 Ma], and the divergence time between *Tamarix* and *Myricaria* was ca. 25.48 Ma (9.87–43.06 Ma). Thus, we used these two related times as normal distribution priors to calibrate the divergence times within *R. songarica*. In the three independent runs of BEAST, the MCMC length was set to 80 million generations, and all parameter values were recorded every 8000 generations. According to the analyses in Tracer version 1.7 (Rambaut et al., 2018), these runs converged to stationary distributions, and the effective sample sizes for all parameters were sufficient. Subsequently, the first 20% of the trees recorded in each run were discarded as burn‐in, while the remaining trees were combined. Finally, the maximum clade credibility tree was constructed using TreeAnnotator version 1.8.0 (Drummond et al., 2012).

We also used the sampled trees and the consensus tree from the BEAST analysis to reconstruct the ancestral state of *R. songarica*. All these trees and the current distribution data were uploaded to RASP version 4.3 (Yu et al., 2020), and the outgroup species were removed. The Statistical Dispersal‐Vicariance model was chosen to analyze these data. A maximum of four areas were allowed at each node, and other parameters were set to default.

### 2.6 Gene flow estimation and demographic history inference

To visualize the geographic pattern of genetic barriers among *R. songarica* populations, bootstrap matrix analyses were conducted using BARRIER version 2.2 (Manni et al., 2004). First, the chlorotypes (or ribotypes) within each population were resampled using PopTools version 3.2.5 (www.bioquest.org) to generate 100 datasets. Then, 100 pairwise *F*_ST_ matrices were calculated from these bootstrap datasets. Finally, genetic barriers between populations were calculated using BARRIER based on the pairwise *F*_ST_ matrices and the geographic locations of the populations.

Based on the cpDNA concatenated sequences, the historical dynamics of maternal gene flow and population size for each genetic group were further estimated using Bayesian inference in MIGRATE‐N version 3.6.11 (Beerli & Palczewski, 2010). Following a previous study (Shi et al., 2020), we set the prior distributions of theta and gene flow (*M*) parameters as uniform distributions between 0 and 0.1 and between 0 and 20,000, respectively. This simulation used one long chain and was run independently 10 times. For static heating, the temperature of parallel chains was set to 1.0, 1.5, 3.0, and 10,000, respectively. In each run, a total of 1 × 10^6^ genealogies were recorded by sampling every 100 steps, and the initial 1 × 10^5^ genealogies were discarded as burn‐in. To calculate the absolute time, we used the mutation rate for *R. songarica* plastome obtained from BEAST analyses, which is 8.96 × 10^−10^ substitutions per site per year (s/s/y). Based on field observations, the generation time of this shrub is estimated to be approximately 3 years (Shi et al., 2020). Additionally, we performed neutrality tests using Arlequin to detect changes in the population size on cpDNA.

## 3 RESULTS

### 3.1 Phylogeny and geographic distribution of chlorotypes

The five cpDNA sequences from 243 *R. songarica* individuals were aligned and concatenated into a data matrix of 3725 bp in length. A total of 37 chlorotypes were identified from this matrix based on 40 nucleotide substitutions and five InDels (Tables 1 and S3). The GuD group had the lowest number of chlorotypes compared to other genetic groups, with 6 of its 11 populations containing only one chlorotype (Table 1). However, the GuD group had the highest nucleotide diversity (*π* = 0.001) and relatively high haplotype diversity (*H*d = 0.653).

**FIGURE 2:**
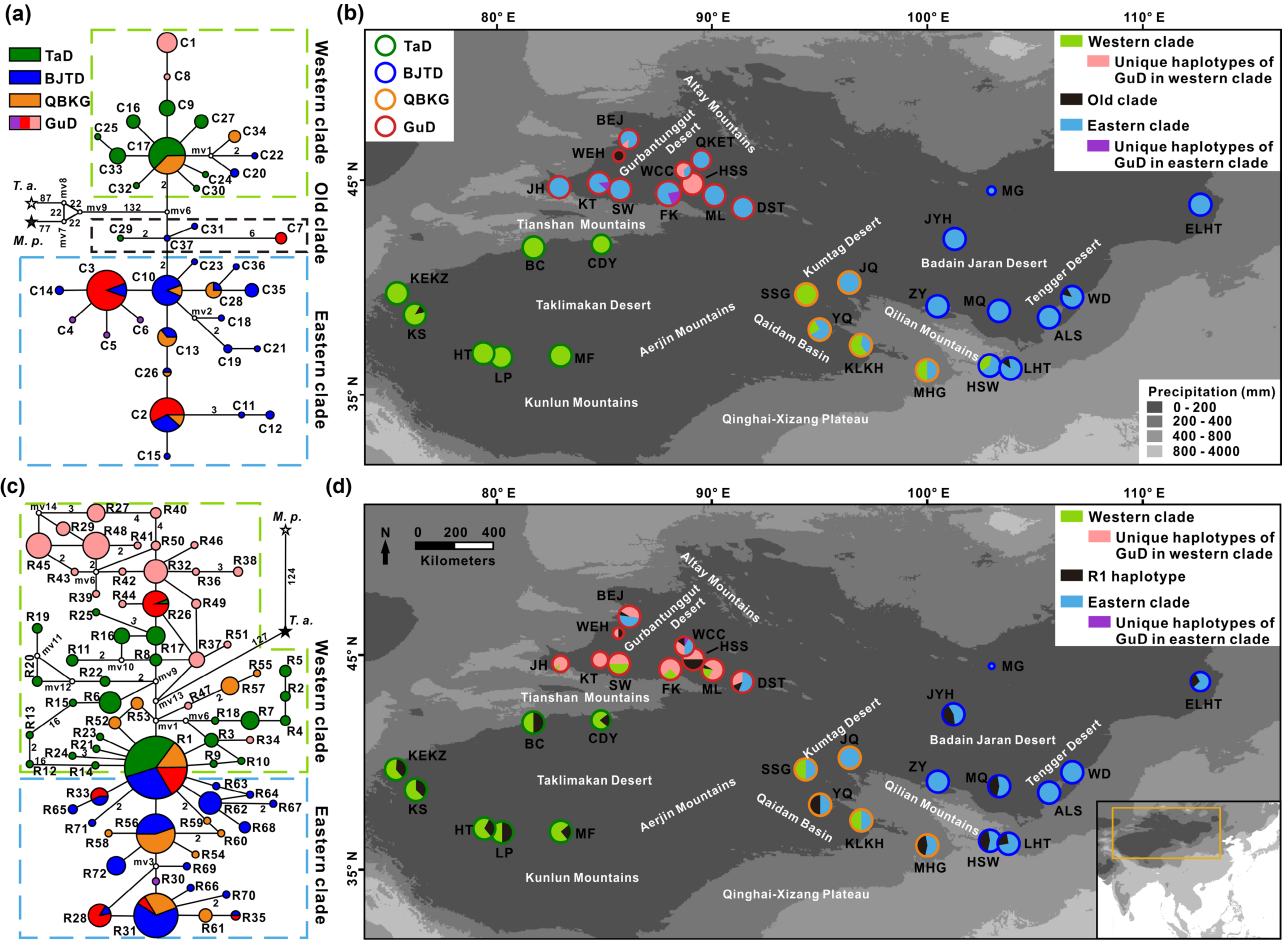
Phylogenetic topologies and geographical distributions of cpDNA (a,b) and nrITS (c,d) haplotypes of *R. songarica*. (a,c) Each pie chart represents one haplotype. The size of the pie chart corresponds to the number of haplotypes. The proportion of fill colors in the pie chart indicates the ratio of the number of corresponding haplotypes in different genetic groups. The stars represent the outgroup haplotypes. The number near the line indicates the number of base differences between two adjacent haplotypes, and only one base difference is not labeled. *M. p*., *Myricaria pulcherrima*; *T. a*., *Tamarix amplexicaulis*. (b,d) Each pie chart corresponds to one population. The size of the pie chart corresponds to the sample size of the population. The fill color in the pie chart corresponds to the grouping information of the haplotypes contained in the population. The ratio of colors represents the proportion of the number of haplotypes in the corresponding clades.

In the genealogical topology, the chlorotypes of *R. songarica* could be classified into three clades (Figure 2a). The western clade included almost all the chlorotypes distributed in the western lineage (the TaD group), while the eastern clade contained most of the chlorotypes from the eastern lineage (the BJTD and QBKG groups). Interestingly, the chlorotypes from the GuD group were asymmetrically distributed in the western and eastern clades and were derived, except for the chlorotype C7, which was directly related to the ancestral chlorotype C37. These phylogenetic relationships among chlorotypes were well supported by the maximum likelihood tree and the BI tree (Figure S1). Furthermore, the geographic distribution of chlorotypes from the three clades showed that the origin of chlorotypes varied widely among the populations of the GuD group (Figure 2b). The WEH population had only the ancestral chlorotype C7; the HSS population possessed the western clade chlorotype; the WCC and BEJ populations had chlorotypes from both the eastern and western clades; while other GuD populations were dominated by the eastern clade chlorotypes. These findings indicated that the GuD group exhibited strong genetic structures in cpDNA.

### 3.2 Phylogeny and geographic distribution of ribotypes

The nrITS fragment of the new samples was sequenced, but no high‐quality sequence was obtained from the QKET population. Based on the aligned nrITS sequences of 662 bp in length, we identified 59 nucleotide substitutions and 72 ribotypes in the remaining 31 populations (Tables S4 and S5). Among the four genetic groups, the GuD group had not only the most ribotypes but also the highest nucleotide diversity (*π* = 0.009) and haplotype diversity (*H*d = 0.925).

Similar to the chlorotypes, the ribotypes from the western and eastern genetic lineages were roughly divided into the western and eastern clades in the genealogical network (Figure 2c). Most of the ribotypes found in the GuD lineage were derived from these clades. In contrast to the chlorotypes, the GuD group had far fewer ribotypes from the eastern clade than from the western clade. Additionally, the ribotype R1 was observed in all genetic groups. Based on the clustering information, the ribotypes were then projected onto a geographic map (Figure 2d). Contrary to the chlorotype distributions, all ribotypes distributed in the JH, KT, SW, ML, and FK populations of the GuD group belonged to the western clade. Moreover, in the GuD group, ribotypes from the eastern clade were found only in the WCC, BEJ, and DST populations, and the ancestral ribotype R1 also occurred in these populations, as well as in the WEH, HSS, and ML populations.

In addition, the pairwise genetic differentiation (*F*_ST_) between the GuD and BJTD genetic groups on cpDNA (0.072) was much lower than that between the GuD and TaD groups (0.638; Table 2). However, we detected the highest *F*_ST_ value (0.406) between the GuD and BJTD groups on nrITS and a lower *F*_ST_ value (0.260) between the GuD and TaD groups.

**TABLE 2:** Pairwise genetic differentiation between genetic groups estimated from cpDNA (lower part) and nrITS (upper part) dataset of *Reaumuria songarica*.

	BJTD	TaD	QBKG	GuD
BJTD	–	0.326***	0.032**	0.406***
TaD	0.790***	–	0.277***	0.260***
QBKG	0.274***	0.456***	–	0.356***
GuD	0.072***	0.638***	0.149***	–

**p < .01;

***p < .001.

In summary, some populations of the GuD lineage possessed only chlorotypes derived from the eastern lineage and ribotypes derived from the western lineage, whereas others experienced genetic admixture of these two lineages on nrITS and/or cpDNA. However, the WEH population had ancient cpDNA and nrITS haplotypes. These findings demonstrated that most populations of the GuD lineage not only originated from hybridization between the eastern and western lineages but may also be highly genetically differentiated.

### 3.3 Phylogeographic structure of the GuD lineage

The total gene diversity (*H*_T_) of all *R. songarica* populations was far higher than the average gene diversity within populations (*H*_S_) on cpDNA (*H*_T_ = 0.920, *H*_S_ = 0.405) and nrITS (*H*_T_ = 0.921, *H*_S_ = 0.701). Moreover, the *N*_ST_ values of overall populations were significantly higher than their *G*_ST_ values (Table 3), indicating a significant phylogeographic structure within the species range of *R. songarica*. This distinct phylogeographic structure was further supported by the AMOVA results, which showed that the molecular variation among the four genetic groups accounted for 43.45% and 29.73% of the total variation in cpDNA and nrITS, respectively, and the *F*_ST_ values of all populations were as high as 0.758 on cpDNA and 0.471 on nrITS (Tables 4 and S6).

**TABLE 3:** Estimates of genetic diversity of different genetic groups of *Reaumuria songarica* based on cpDNA and nrITS sequences.

Dataset	Groups	*H*_S_	*H*_T_	*G*_ST_	*N*_ST_	*V*_S_	*V*_T_
cpDNA	GuD	0.196 (0.071)	0.746 (0.079)	0.738 (0.099)	0.799 (0.109)	0.150 (0.082)	0.750 (0.171)
	TaD	0.321 (0.091)	0.819 (0.076)	0.608 (0.150)	0.599 (0.176)	0.328 (0.126)	0.818 (0.109)
	BJTD	0.701 (0.066)	0.891 (0.051)	0.214 (0.054)	0.478 (0.142)**	0.477 (0.121)**	0.915 (0.223)
	QBKG	0.450 (0.143)	0.877 (0.098)	0.487 (0.155)	0.456 (0.217)	0.474 (0.188)	0.871 (0.084)
	All without GuD	0.515 (0.063)	0.918 (0.027)	0.439 (0.067)	0.672 (0.075)**	0.304 (0.066)**	0.927 (0.088)
	All four groups	0.405 (0.055)	0.920 (0.018)	0.560 (0.059)	0.730 (0.061)**	0.250 (0.053)**	0.925 (0.079)
nrITS	GuD	0.738 (0.046)	0.945 (0.012)	0.219 (0.048)	0.228 (0.028)	0.731 (0.083)	0.946 (0.087)
	TaD	0.758 (0.052)	0.860 (0.033)	0.119 (0.041)	0.199 (0.027)	0.697 (0.068)	0.870 (0.072)
	BJTD	0.647 (0.036)	0.820 (0.040)	0.211 (0.037)	0.206 (0.098)	0.651 (0.094)	0.821 (0.058)
	QBKG	0.643 (0.029)	0.871 (0.032)	0.261 (0.026)	0.362 (0.068)	0.567 (0.065)	0.889 (0.094)
	All without GuD	0.683 (0.026)	0.881 (0.021)	0.225 (0.024)	0.361 (0.026)**	0.566 (0.053)	0.887 (0.071)
	All four groups	0.701 (0.023)	0.921 (0.015)	0.240 (0.022)	0.421 (0.040)**	0.537 (0.049)*	0.926 (0.065)

*Note*: The number in the parenthesis is the standard error.

Abbreviations: *G*_ST_, interpopulation differentiation; *H*_S_ and *V*_S_ (multiloci), average gene diversity within populations; *H*_T_ and *V*_T_ (multiloci), total gene diversity; *N*_ST_, number of substitution types.

*p < .05;

**p < .01.

**TABLE 4:** Analyses of the molecular variance (AMOVA) of cpDNA sequences for different groups of *Reaumuria songarica*.

Groups	Source of variation	Degree of freedom	Sum of squares	Variance component	Percentage of variation (%)	Fixation index
All four groups	Among groups	3	457.849	2.316 Va	43.45	*F*_CT_ = 0.434***
	Among populations within groups	28	401.756	1.727 Vb	32.39	*F*_SC_ = 0.573***
	Within populations	211	271.843	1.288 Vc	24.16	*F*_ST_ = 0.758***
	Total	242	1131.449	5.332		
BJTD, TaD, QBKG, GuD‐S, and GuD‐N	Among groups	4	545.741	2.595 Va	49.42	*F*_CT_ = 0.494***
	Among populations within groups	27	313.865	1.367 Vb	26.04	*F*_SC_ = 0.515***
	Within populations	211	271.843	1.288 Vc	24.54	*F*_ST_ = 0.755***
	Total	242	1131.449	5.25		
All without GuD	Among groups	2	375.765	3.389 Va	58.38	*F*_CT_ = 0.584***
	Among populations within groups	18	152.936	0.909 Vb	15.67	*F*_SC_ = 0.376***
	Within populations	141	212.417	1.507 Vc	25.95	*F*_ST_ = 0.741***
	Total	161	741.117	5.805		
BJTD	Among populations	8	53.749	0.689 Va	29.81	*F*_ST_ = 0.298***
	within populations	58	94.042	1.621 Vb	70.19	
	Total	66	147.791	2.31		
TaD	Among populations	6	20.986	0.370 Va	38.62	*F*_ST_ = 0.386***
	within populations	48	28.250	0.589 Vb	61.38	
	Total	54	49.236	0.959		
QBKG	Among populations	4	78.200	2.122 Va	45.18	*F*_ST_ = 0.452***
	within populations	35	90.125	2.575 Vb	54.82	
	Total	39	168.325	4.697		
GuD	Among populations	10	248.820	3.274 Va	79.41	*F*_ST_ = 0.794***
	within populations	70	59.427	0.849 Vb	20.59	
	Total	80	308.247	4.123		

*Note*: In subsequent analyses, the GuD group was subdivided into two groups, GuD‐S and GuD‐N.

Abbreviations: *F*_CT_, correlation of haplotypes within groups relative to total; *F*_SC_, correlation within populations relative to groups; *F*_ST_, correlation within populations relative to total.

***p < .001.

Although the *N*_ST_ values of the GuD lineage were slightly higher than its *G*_ST_ values (*p* > .05), the *H*_T_ values of this lineage were much greater than its *H*_S_ values (*H*_T_ = 0.746 and *H*_S_ = 0.196 for cpDNA; *H*_T_ = 0.945 and *H*_S_ = 0.738 for nrITS) (Table 3). Meanwhile, the AMOVA analyses showed that 79.41% of the total variation of cpDNA within the GuD lineage was distributed among populations, but the molecular variation of cpDNA within other genetic groups was mainly distributed within populations (Table 4). These findings suggested a distinct phylogeographic structure within the GuD lineage.

To further determine the optimal population grouping within the GuD lineage, we performed SAMOVA on the cpDNA datasets. When all *R. songarica* populations were analyzed, the *F*_CT_ values peaked at *K* = 6 (Figure S2a). For *K* greater than two, one of the groups contained only the WEH population. When *K* = 6, most of the populations from the GuD and eastern lineages formed one group, all populations from the western lineage clustered with the SSG population, the HSS and WCC populations from the GuD lineage formed one group, the KLKH, MHG, and HSW populations formed another group, and the last two groups contained the WEH and MG populations, respectively. Furthermore, the analysis considering only the GuD lineage showed that the *F*_CT_ value was highest at *K* = 5 (Figure S2b). In this optimal population grouping, the ML, FK, JH, KT, and SW populations near the Tianshan Mountains formed one group, the QKET, DST, and BEJ populations clustered into a group, and the HSS, WCC, and WEH populations were separated into the other three groups (Table S7).

**FIGURE 3:**
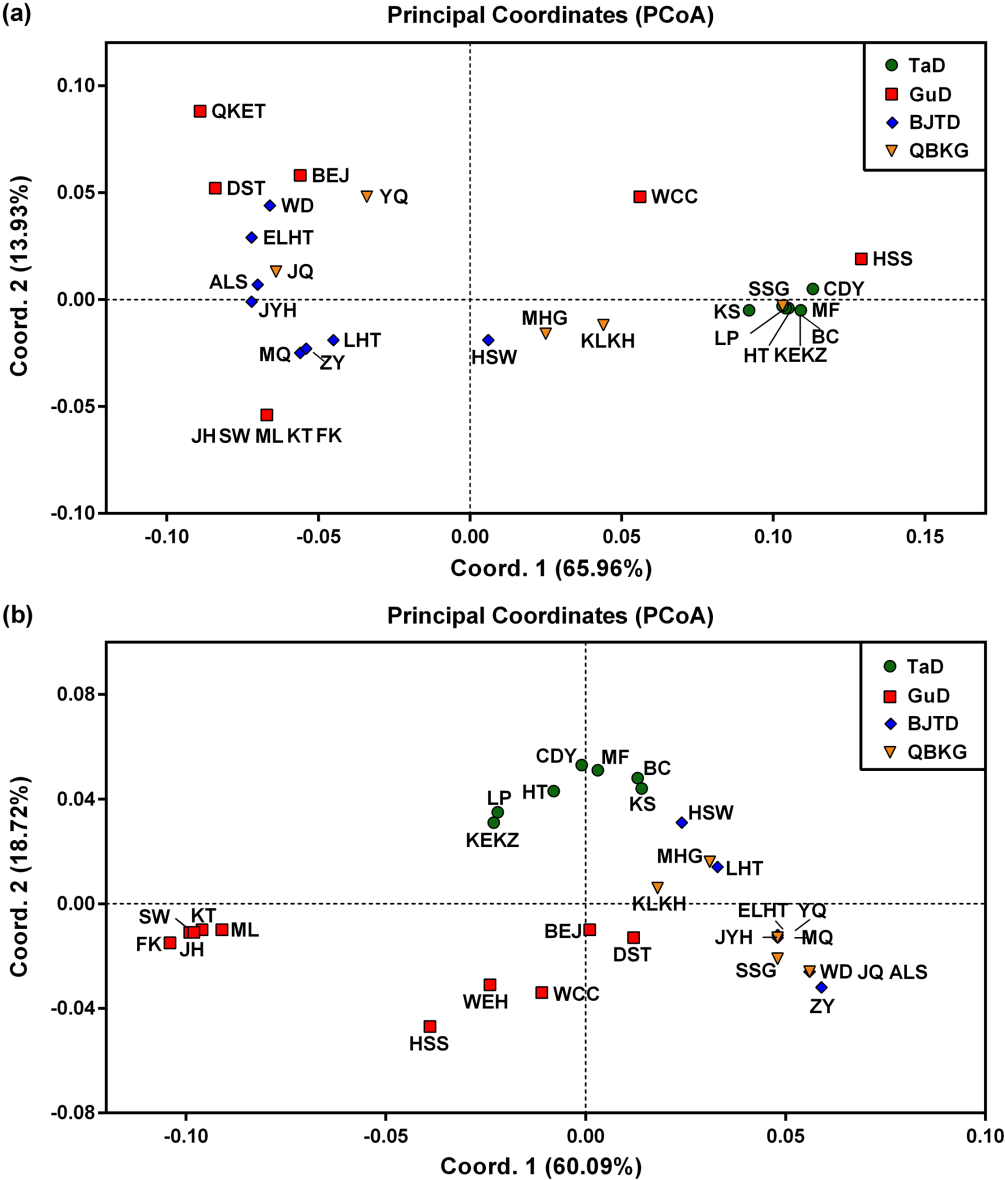
Plots of Principal Coordinates analysis (PCoA) based on cpDNA (a) and nrITS (b) data of *R. songarica*.

In addition, PCoA results showed that in the GuD lineage, the five populations near the Tianshan Mountains were clustered together and strongly genetically differentiated from the other populations (Figure 3). Notably, these five populations were distinctly separated from the eastern and western lineages on nrITS (Figure 3b). Moreover, in the analysis based on cpDNA, the QKET, DST, and BEJ populations of the GuD lineage had close genetic relationships, and the genetic distances between these populations and the HSS (or WCC) population were even close to those between the eastern and western lineages (Figure 3a).

Taken together, the ML, FK, SW, KT, and JH populations had similar genetic components and were strongly differentiated from other populations of the GuD lineage. Therefore, these five populations can be classified into a single genetic group of *R. songarica*. This group of the GuD lineage was designated GuD‐S because it was located at the southern margin of the Gurbantunggut Desert near the Tianshan Mountains.

### 3.4 Genetic barrier and gene flow

The MIGRATE‐N results showed continuous maternal gene flow between all pairs of *R. songarica* lineages (Figure S3), suggesting that these lineages were not completely genetically isolated. The gene flow between most lineages fluctuated dramatically and increased after the last interglacial period (0.13 Ma). Our results also indicated that the GuD lineage received more gene flow from the eastern lineage than from the western lineage. Furthermore, the bidirectional migration between the GuD lineage and the BJTD group was markedly asymmetric.

**FIGURE 4:**
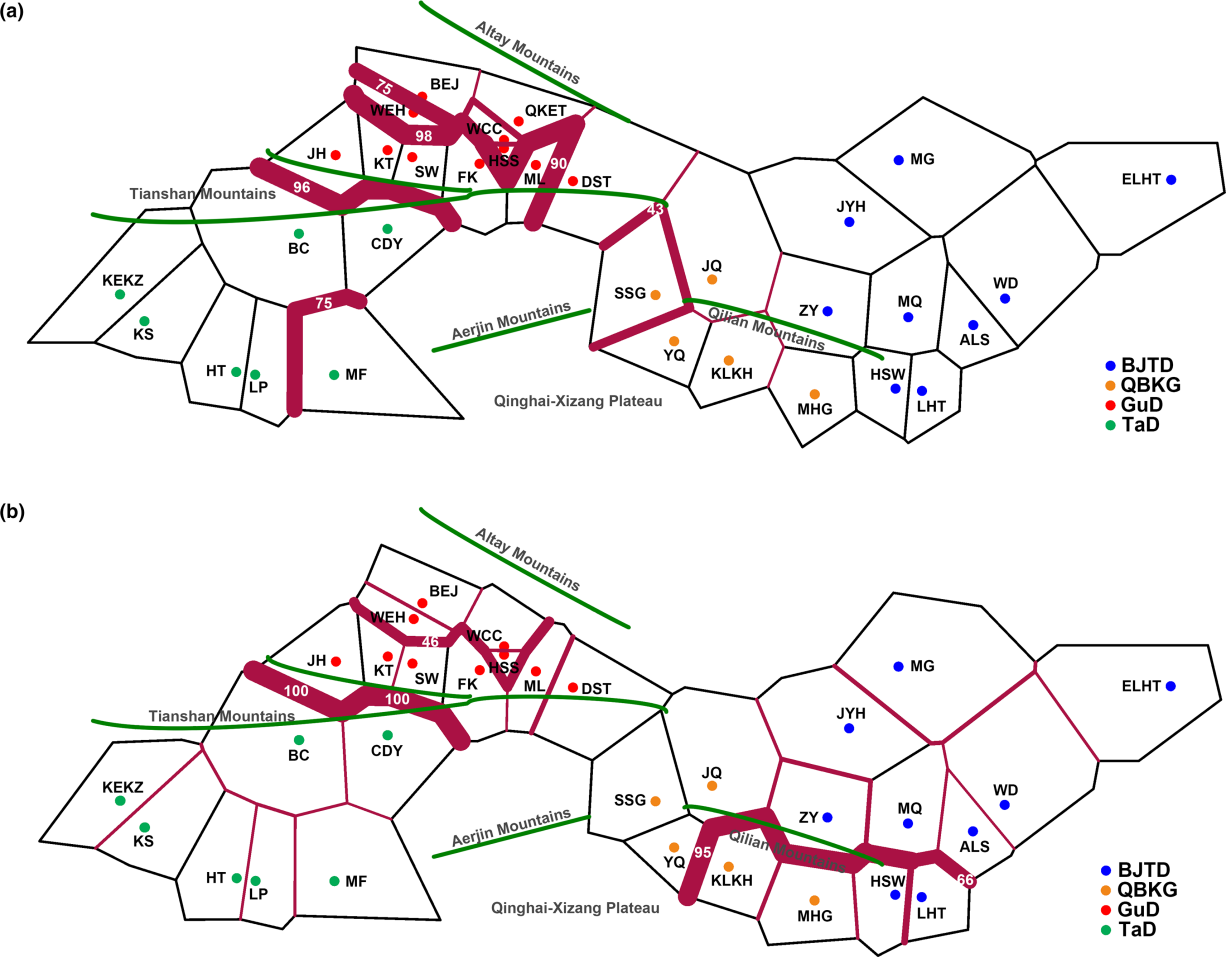
Geographic patterns of genetic barriers among *R. songarica* populations based on cpDNA (a) and nrITS (b) data. Genetic barriers between populations are represented by red lines, and the thickness of these lines (bootstrap value) represents the strength of the genetic barrier. Mountains are represented by green lines.

To further detect the geographic pattern of contemporary genetic discontinuity among *R. songarica* populations, we conducted genetic barrier analyses. The results indicated that the highest level of the genetic barrier occurred between the GuD‐S group and the western lineage on cpDNA and nrITS, which was caused by a geological barrier, the Tianshan Mountains (Figure 4). However, much weaker genetic barriers were found between the eastern lineage and some other populations of the GuD lineage. Within the GuD lineage, the strength of the genetic barrier on cpDNA between the GuD‐S group and the other populations was unexpectedly as high as that between the GuD‐S group and the western lineage (Figure 4a). Even on the biparentally inherited nrITS, there was still a moderate level of genetic barrier between the GuD‐S group and other GuD populations (Figure 4b).

### 3.5 Divergence times and ancestral regions of genetic lineages

**FIGURE 5:**
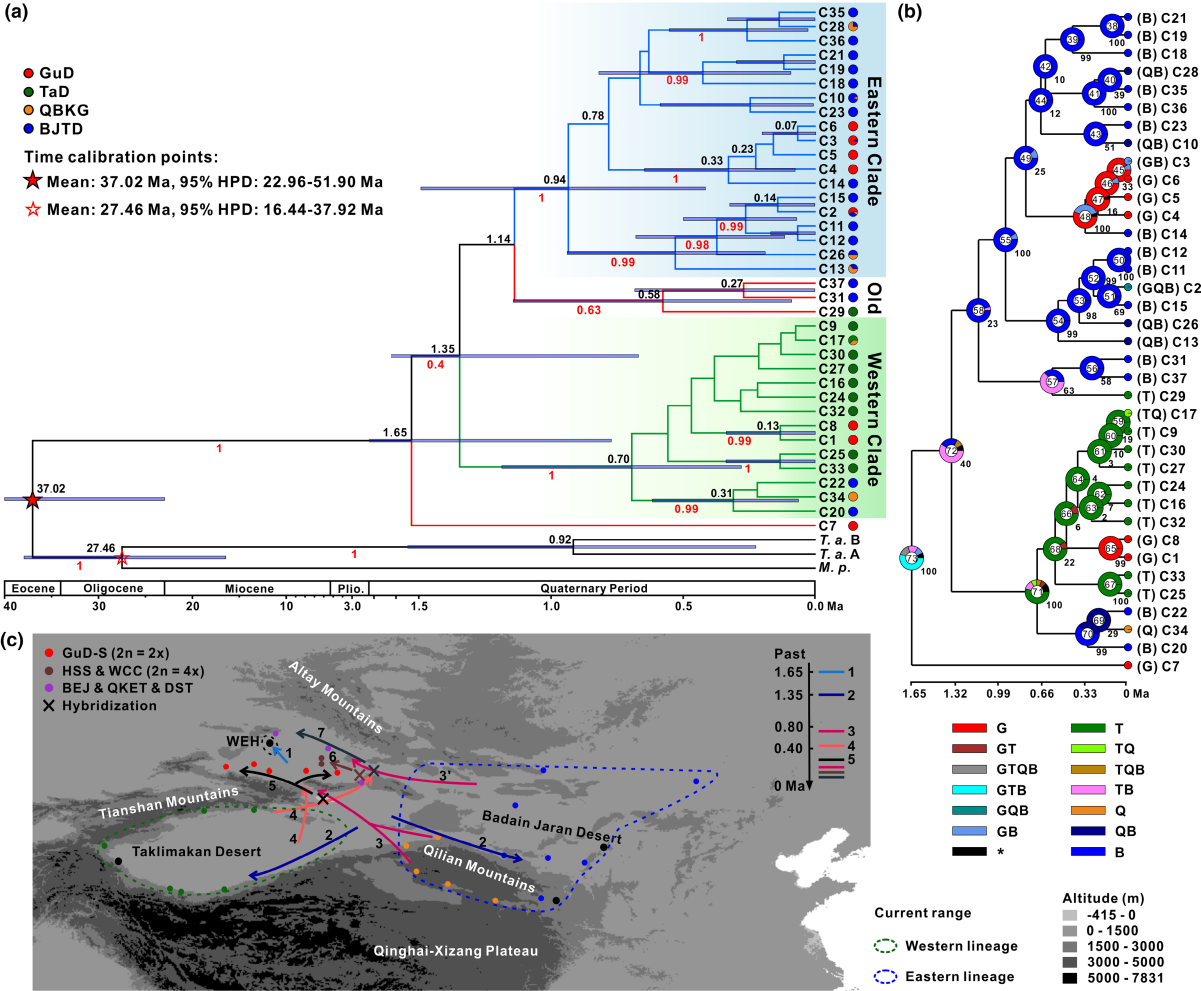
Reconstruction of the spatial and temporal evolutionary history of *R. songarica* based on cpDNA haplotypes. (a) The maximum clade credibility tree constructed using BEAST. The black number near the node is the median node age, and the length of the purple line represents the 95% highest posterior density (HPD) of the node age. The red numbers are the posterior probabilities. The different colors in the pie chart on the right side represent different genetic groups, and the ratio of colors indicates the ratio of the number of corresponding haplotypes in different groups. Plio., Pliocene. (b) Reconstruction of ancestral regions using RASP. The size of each color in the pie chart represents the probability that the ancestor is distributed in the corresponding region. G, GuD; T, TaD; Q, QBKG; B, BJTD. Areas with a probability of less than 5% are marked with an asterisk. (c) Schematic diagram of the spatial and temporal evolutionary history inferred from (a,b). The lines represent the dispersal routes of the genetic groups. 1, the WEH population diverged from the common ancestor. 2, the ancestor further diverged into the eastern and western lineages. 3 and 4, the eastern and western lineages spread successively into northern Xinjiang. 5, 6, and 7, the GuD‐S group and other hybrids probably migrated from their places of origin to their current ranges.

Based on the chlorotypes, the BEAST analysis showed that the estimated age of the crown of Tamaricaceae was approximately 37.02 Ma (95% HPD, 22.96–51.90 Ma), and the divergence time between *Tamarix* and *Myricaria* was estimated to be 27.46 Ma (16.44–37.92 Ma) (Figure 5a). These two divergence times are consistent with the ages of the corresponding nodes calibrated from complete chloroplast genomes (Yao et al., 2019), indicating a high degree of credibility for our result. The chlorotype C7, unique to the GuD lineage, diverged from the common ancestor at around 1.65 Ma (0.77–2.62 Ma), which was earlier than the divergence time between the eastern and western clades of *R. songarica* chlorotypes (1.35 Ma [0.67–2.11 Ma]). Moreover, the crown age of the eastern clade (0.94 Ma [0.42–1.49 Ma]) was older than that of the western clade (0.7 Ma [0.28–1.19 Ma]). The chlorotypes of the GuD lineage, except for C7, were clustered into three minimum clades, and the stem age of these clades was younger than 0.5 Ma. Additionally, the result of RASP suggested that the ancestor of *R. songarica* likely had a wide distribution across the range of its eastern, western, and GuD lineages (Figure 5b). However, except for C7 from the WEH population, the ancestor of the chlorotypes distributed in the GuD lineage dispersed multiple times into northern Xinjiang from the western and eastern lineages, respectively. The possible spatial and temporal dispersal routes of the ancestral parents of the northern Xinjiang populations are shown in Figure 5c.

### 3.6 Demographic history of *Reaumuria songarica*

**FIGURE 6:**
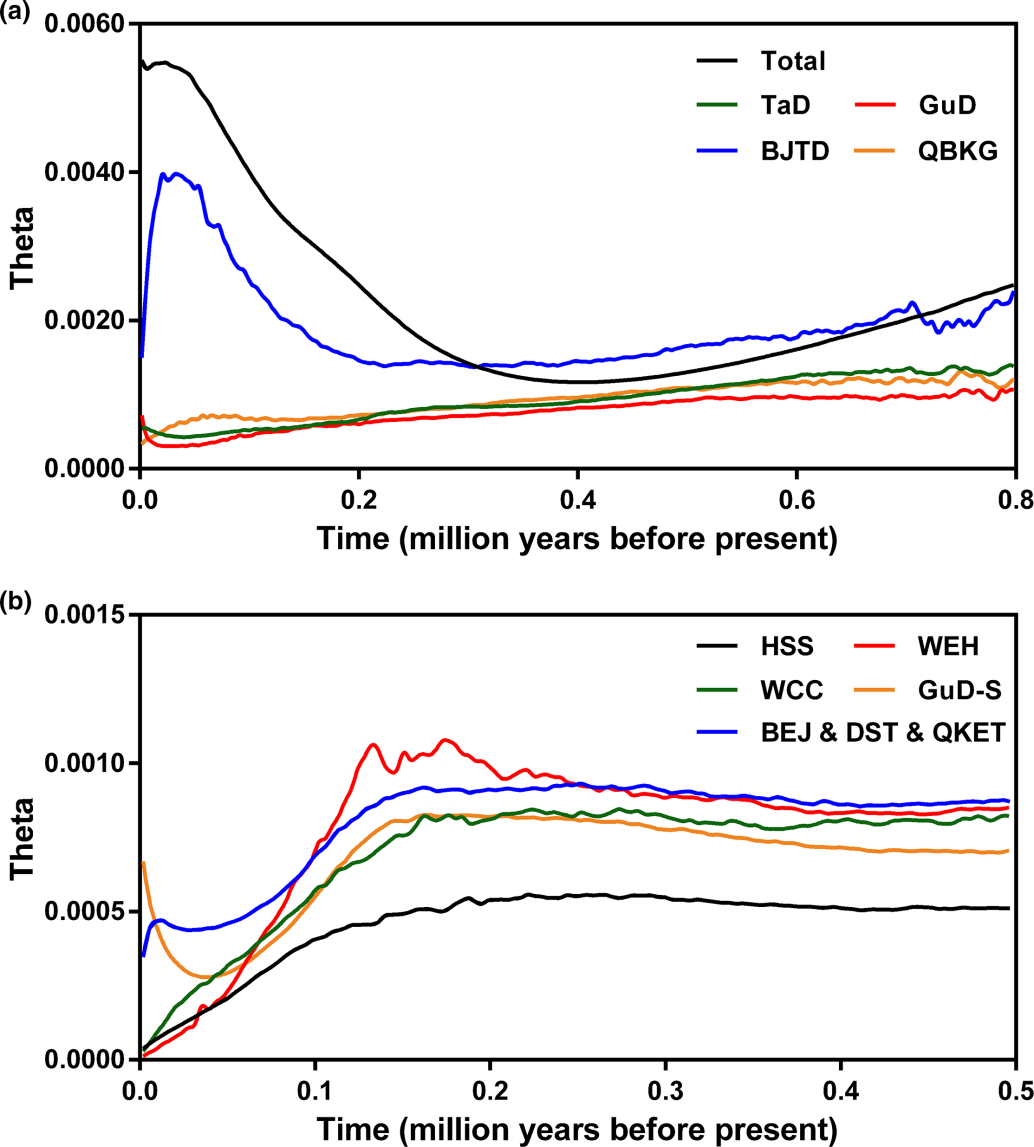
Skyline plots for the effective population size (*N*e) of *R. songarica* based on cpDNA data. (a) Changes in the *N*e of *R. songarica* and its four groups. (b) Changes in the *N*e of different groups of the GuD lineage. Theta (θ) = 2μ*N*e.

The neutral tests on cpDNA showed marginally significant negative values of Fu's *Fs* (−10.59, *p* < .01) and Tajima's *D* (−1.07, *p* < .1) for the entire *R. songarica*, suggesting that this species may have experienced a population expansion (Table S8). The skyline plot further demonstrated that the population size of this species had increased dramatically since ca. 0.4 Ma (Figure 6a). Among the genetic groups, the BJTD group had the largest population size, which started to increase at approximately 0.2 Ma and then decreased sharply from ca. 0.03 Ma, suggesting that the BJTD group experienced a significant bottleneck. In contrast, the population size of the GuD lineage declined slowly until the last glacial maximum (ca. 0.03 Ma) and increased considerably thereafter (Figure 6a). Within the GuD lineage, only the GuD‐S group underwent a population expansion since ca. 0.03 Ma (Figure 6b), which was consistent with the significant negative values of its Fu's *Fs* and Tajima's *D* (Table S8). However, in other hypothetical groups of the GuD lineage, the population sizes began to decline consistently during the last interglacial period (Figure 6b), which could be caused by the greatly enhanced gene flow from other lineages to the GuD lineage since this period. It should be noted that the estimated population sizes of these groups of the GuD lineage may differ slightly from the true values due to limited genetic variation in some or a particular population used in these analyses.

## 4 DISCUSSION

### 4.1 The ancient origin of the WEH population in northern Xinjiang

Based on the variation patterns of cpDNA sequences and nuclear loci, previous studies have inferred that the *R. songarica* populations in northern Xinjiang originated from hybrids (Shi et al., 2020; Yin et al., 2015). However, this view may be biased because the northern Xinjiang populations analyzed in these studies are located only at the southeastern edge of the region. Therefore, we resampled 11 populations that covered the entire range of *R. songarica* in northern Xinjiang and investigated their genetic components. Our results showed that the endemic chlorotype C7 of the WEH population from northern Xinjiang belonged to the ancestral chlorotype, while each individual in this population had the ancestral ribotype R1. Thus, the WEH population has evolved directly from the ancestor of *R. songarica*. Except for this population, the remaining populations in northern Xinjiang originated through hybridization (Figure 2).

It is noteworthy that C7 was not only an ancestral chlorotype but also the haplotype that first diverged from the common ancestor (Figure S1). Based on the BEAST analysis, the divergence time between C7 and the other chlorotypes of *R. songarica* was dated to 1.65 Ma (0.77–2.62 Ma, Figure 5). However, this divergence time is even later than the crown age of the eastern and western lineages (2.96 Ma [1.45–5.5 Ma]) estimated in our previous study (Yin et al., 2015). Upon comparing these two studies, we argue that the conflicting divergence times inferred from the same cpDNA sequences are mainly caused by the different parameters used in the BEAST analysis. Specifically, Yin et al. (2015) selected a conservative mutation rate of 4.87 × 10^−10^ s/s/y for *R. songarica* plastome. However, this rate does not reflect its true mutation rate and is slower than that of most shrubs (Jia et al., 2012; Richardson et al., 2001; Wolfe et al., 1987). In the current analyses, instead of assuming a constant mutation rate, we prudently chose two related diversification times as prior distributions, which were calibrated using a large number of complete plastomes and fossil records (Yao et al., 2019), as detailed in the Materials and Methods section. Our result showed that the estimated age for the divergence between *Tamarix* and *Myricaria* was approximately 27.46 Ma, which predates the oldest fossil record (23 Ma) of *Tamarix* (Zhang et al., 2003). Additionally, the average substitution rate for the *R. songarica* plastome calculated in the current analysis was 8.96 × 10^−10^ s/s/y, which is close to the rates used for other desert shrubs (Jia et al., 2012; Jia & Zhang, 2019; Liang et al., 2018). Therefore, the divergence times among *R. songarica* chlorotypes estimated in this study are more objective.

The divergence time between C7 and other chlorotypes coincides with the fluctuation of the EAMS during the early Pleistocene. According to new evidence from a high‐resolution pollen record, the East Asian Summer Monsoon was stronger between 2.2 and 2.06 Ma, then weakened stepwise until 1.97 Ma, after which it gradually strengthened again between 1.97 and 1.7 Ma (Hui et al., 2021). Numerous studies have demonstrated that climatic oscillations influenced by EAMS dynamics may have increased environmental heterogeneity, fragmented the habitat of widespread desert plants in northwestern China, and further promoted their intraspecific differentiation, such as *Nitraria tangutorum* and *Gymnocarpos przewalskii* (Jia & Zhang, 2019; Yin et al., 2020). Indeed, the potential distribution range of *R. songarica* under both glacial and interglacial climates is much smaller and more fragmented than that under the current climatic conditions (Yin et al., 2015). Taken together, we believe that the fluctuation of the EAMS is likely to be one of the main drivers of the divergence between the WEH population and other populations of *R. songarica*.

Compared to other desert floras in China, the formation of the Junggar Basin flora is the most recent (Liu, 1995). However, this does not mean that all the extant species in this region migrated from the surrounding deserts (Zhang & Chen, 2002). For example, based on multiple molecular evidence, Qian et al. (2016, 2021) found that the annual pioneer herb *Agriophyllum squarrosum* originated in the Gurbantunggut Desert and gradually colonized other Chinese deserts. Another study on the desert tree *Populus euphratica* demonstrated that its populations in northern and southern Xinjiang are clearly divergent, and the diversification of chlorotypes from northern Xinjiang is earlier than that of chlorotypes from southern Xinjiang (Zeng et al., 2018). In this study, the WEH population in northern Xinjiang was an ancient population, and its formation preceded the divergence between *R. songarica* populations in other regions. These phylogeographic data provide new evidence that desertification in northern Xinjiang started before 1.2 Ma (Shi et al., 2006). However, after about 0.52 Ma, several glaciations of different scales occurred in the Tianshan Mountains (Zhao et al., 2015), and these glaciers probably destroyed some of the early desert flora in the Junggar Basin (Liu, 1995). Therefore, the evolutionary origin of species diversity in the Junggar Basin flora is more complex than previously known.

### 4.2 Different types of hybrids in the GuD lineage

Our results revealed that the hybrid populations from the GuD lineage had different ancestral parents. The five populations in the GuD‐S group had similar genetic components, with the eastern lineage as their female parent and the western lineage as their male parent. However, the western and eastern lineages were the ancestral female parents of the WCC and HSS populations, and the BEJ, QKET, and DST populations, respectively; these populations from the GuD lineage experienced genetic admixture of the parental lineages at nrITS (Figure 2). Furthermore, the divergence time of the chlorotypes (0.23 Ma; C3 to C6) possessed by the GuD‐S group was markedly different from that of the chlorotypes (0.13 Ma; C1 and C8) unique to other GuD populations (Figure 5). Notably, the DNA 1C‐value of the different populations indicated that the GuD‐S group and the eastern and western lineages are diploid, whereas the WCC and HSS populations are tetraploid (Fan et al., 2021). These allopolyploid populations inhabit more hostile environments compared to the homoploid hybrid populations, and polyploidization may have facilitated their adaptation to these harsh conditions (Fan et al., 2021). A previous study based on multiple nuclear loci suggested that the GuD lineage originated from multiple hybridizations between the eastern and western lineages (Shi et al., 2020). Our new data, as mentioned above, further indicate that the derived populations of the GuD lineage originated from different hybridization events (Figure 5c). Similarly, in different hybridization events, *Pinus tabuliformis* and *P. yunnanensis* served as the initial female parents of different *P. densata* populations, respectively (Gao et al., 2012; Song et al., 2003).

Two species with the same ploidy can produce one or more new homoploid hybrid species through multiple hybridization events (Ma et al., 2006; Rieseberg, 1991; Schwarzbach & Rieseberg, 2002). However, their hybrid offspring may also only form hybrid zones (Jiang et al., 2016). Here, several lines of evidence further support the existence of a significant phylogeographic structure within the GuD lineage. First, for both cpDNA and nrITS, the total gene diversity of the GuD lineage was much higher than the average gene diversity within its populations (Table 3). The AMOVA result also indicated that the molecular variation of cpDNA within the GuD lineage was mainly distributed among populations (Table 4). Second, using cpDNA datasets for spatial genetic structure analyses, the populations of the GuD lineage could be classified into the GuD‐S group and four other putative groups (Table S7). Surprisingly, the genetic distance between these groups of the GuD lineage was even close to that between the eastern and western lineages (Figure 3). Third, the BARRIER results also showed strong genetic barriers on cpDNA and nrITS between the GuD‐S group and other GuD populations (Figure 4). Taken together, these results suggest that the GuD lineage has diverged into distinct genetic groups, while their populations have undergone different and complex demographic histories.

Compared with its parental lineages, the GuD lineage occupies a new ecological niche with lower winter temperatures and has significantly larger leaf sizes adapted to this niche (Fan et al., 2020). Therefore, the GuD lineage may be an incipient species arising from ecological speciation (Shi et al., 2020). Our findings indicate that the highest level of genetic barrier occurs between the GuD‐S group and its parental lineages (Figures 3 and 4). Meanwhile, the GuD‐S group includes three of the four populations of the GuD lineage used by Shi et al. (2020). Therefore, we conclude that only the GuD‐S group is a homoploid hybrid species at an early stage of ecological speciation. In addition, other populations of the GuD lineage have different ancestral female parents and may have different ploidy levels. However, these populations showed a genetic admixture of the eastern and western lineages at nrITS (Figure 2d), suggesting that they may be allopolyploid populations and/or hybrid zones. This is also observed in *Pinus densata*, whose ancient hybrid zone has distinct mitochondrial haplotypes found in the two parental species (Gao et al., 2012; Wang et al., 2011). To simplify the study of the genetic mechanisms underlying speciation in *R. songarica*, we consolidated these other GuD populations into a group called GuD‐N. This population grouping of the GuD lineage is supported by the optimal population structure inferred from restriction‐site associated DNA sequencing (RAD‐seq) data from *R. songarica* (Fan et al., unpublished data), which also revealed the same nuclear genetic composition for the QKET and DST populations.

### 4.3 Driving factors of the evolution of *Reaumuria songarica* in northern Xinjiang

In addition to climatic fluctuations, past geological events have profoundly influenced the geographic distribution and evolutionary history of most extant plant species in the Northern Hemisphere (Ding et al., 2020; Hewitt, 2000). In ACA, climate change (e.g., the development of the EAMS) and paleogeological events (e.g., the uplift of the QXP) have combined to drive the progressive aridification of the region, thereby providing new ecological niches for the ancestors of desert plants (An et al., 2001; Guo et al., 2002; Miao et al., 2012). Several phylogeographic studies have found that these factors have caused habitat movement and fragmentation, increasing environmental heterogeneity among different populations; these consequences may further promote intraspecific divergence, hybridization, and speciation in desert plants (Meng et al., 2015; Qian et al., 2021; Shi et al., 2020). For example, the diversification and dispersal processes of *Reaumuria* species adapted to arid environments in Asia were mainly influenced by the uplift of the QXP and global cooling during the Eocene–Oligocene Transition (Zhang et al., 2014). Such studies also suggest that desert plants endemic to ACA may have responded differently to these geological events and climate change (Jia et al., 2020; Jia & Zhang, 2019; Qian et al., 2016).

Available empirical and theoretical evidence suggests that ecological selection plays an important role in promoting HHS (Buerkle et al., 2000; Gross & Rieseberg, 2005; Wang et al., 2021). For instance, *Pinus densata* is distributed in high‐altitude habitats inaccessible to its parental species, and the divergent selection among these species is a major driver of the speciation of *P. densata* (Zhao et al., 2014). Our results suggest that *R. songarica* diverged into the eastern and western lineages at 1.35 Ma (0.67–2.11 Ma). During this period, the Kunlun‐Yellow River tectonic movement (1.1–0.6 Ma) uplifted the QXP to over 3000 m, leading to a colder and drier climate in northwestern China, intensifying the East Asian Winter Monsoon (EAWM), and increasing climatic differences among arid regions (Cui et al., 1998; Meng et al., 2011; Zheng et al., 2002). As a result, geographic isolation and ecological differentiation occurred between the eastern and western lineages, driving their genetic divergence (Shi et al., 2020). Additionally, these geological events and climatic fluctuations intensified the aridification of northern Xinjiang and led to the formation of the main body of the Gurbantunggut Desert during 1.2–0.8 Ma (Shi et al., 2006). Thus, the Gurbantunggut Desert represents a new potential habitat for the eastern and western lineages. The change in loess grain size revealed that this desert apparently expanded at about 0.65 and 0.6 Ma, followed by a gradual yet highly fluctuating expansion between 0.4 and 0.13 Ma (Shi et al., 2006). These time points coincide with the onset of the expansion of the eastern (0.6–0.55 Ma) and western (0.25–0.2 Ma) lineages driven by a stronger EAWM (Shi et al., 2020). Moreover, the crown age of the chlorotypes found in the GuD lineage was also during the expansion period of the Gurbantunggut Desert. Therefore, we suggest that with the multiple expansions of the Gurbantunggut Desert, the eastern and western lineages successively colonized the vicinity of this region and formed different hybrids through multiple contacts. Ecological niche modeling suggested that the Gurbantunggut Desert is an unsuitable area for the eastern and western lineages under current climatic conditions (Shi et al., 2020). This ecological selection may prevent the initial hybrids of *R. songarica* from disappearing in the face of ongoing gene flow from their parental lineages.

After 0.13 Ma, the Gurbantunggut Desert expanded slightly during glacial periods and gradually contracted during interglacial periods (Lu et al., 2013; Shi et al., 2006). However, both the glacial and interglacial climates may have fragmented the potential habitat of *R. songarica* (Yin et al., 2015). The greater genetic distance between the GuD‐S and GuD‐N groups (Figure 3) also suggests that they were once geographically isolated from each other. A similar phenomenon has been observed in *Populus euphratica* populations in northern Xinjiang (Zeng et al., 2018). In addition, the latest deformation (7–5 Ma) of the Tianshan Mountains (Tang et al., 2016) occurred much earlier than the divergence between the eastern and western lineages. Therefore, the current distribution area of the GuD‐S group is unlikely to be the region where the parental lineages originally hybridized. We speculate that the ancestors of the GuD‐S group may have migrated into this area as a result of climate oscillations (Figure 5c). Later, the Tianshan Mountains prevented gene flow from the western lineage, and the dominant westerly circulation (Liu, 2020) may have greatly reduced gene flow from the eastern lineage and the GuD‐N group. These geographic factors, together with ecological selection, provided opportunities for the independent evolution of the GuD‐S group. However, populations of the GuD‐N group, located at the eastern edge of the Gurbantunggut Desert, face continuous gene flow from the eastern lineage, as they are far from the Tianshan Mountains and are more or less affected by the fluctuation of the EAMS (Shi et al., 2020; Yin et al., 2015). This finding could explain why the diploid populations of the GuD‐N group, which are closer to the eastern lineage, contain higher proportions of genetic composition from the eastern lineage (Figure 2d). In summary, we argue that Quaternary climatic oscillations and interregional climatic differences further increased the genetic differentiation between the GuD‐S and the GuD‐N groups.

## 5 CONCLUSION

The asynchronous aridification and fluctuations of different desert regions drove intraspecific divergence and hybridization in *R. songarica*. Our study found that most populations in northern Xinjiang originated from different hybridization events. Only the GuD‐S group, which originated from hybridization between the eastern and western lineages, belongs to the homoploid hybrid lineage. The GuD‐N group, characterized by genetic admixture, may include hybrid zones or tetraploid hybrid populations. Additionally, the WEH population in northern Xinjiang is descended from ancient *R. songarica* and may have survived by growing in glacial refugia. During the Quaternary, the Kunlun‐Yellow River tectonic movement and climatic oscillations led to the multiple expansions and contractions of the Gurbantunggut Desert, providing opportunities for secondary contact or geographic isolation among different *R. songarica* lineages. This is likely to be one of the main reasons for the complex origins of the populations in northern Xinjiang. Our study also shows that geographic isolation, as well as significant ecological differentiation, played an important role in the independent evolution of the GuD‐S group. To further elucidate the genetic mechanism of this early hybrid speciation, we are currently analyzing the RAD‐seq data from *R. songarica* populations.

## AUTHOR CONTRIBUTIONS

**Xingke Fan:** Formal analysis (lead); funding acquisition (supporting); writing – original draft (lead); writing – review and editing (equal). **Xia Yan:** Formal analysis (supporting); funding acquisition (equal); writing – review and editing (equal). **Chaoju Qian:** Data curation (equal); investigation (equal); supervision (equal); writing – review and editing (supporting). **Ibrahim Awuku:** Data curation (equal); writing – review and editing (supporting). **Pengshu Zhao:** Data curation (equal); investigation (equal). **Yuqiu Liao:** Data curation (equal); investigation (equal). **Zhijun Li:** Conceptualization (equal); writing – review and editing (supporting). **Xinrong Li:** Conceptualization (equal); writing – review and editing (supporting). **Xiao‐Fei Ma:** Conceptualization (lead); funding acquisition (equal); writing – original draft (supporting); writing – review and editing (lead).

## CONFLICT OF INTEREST STATEMENT

The authors have no conflict of interest to declare.

## Supporting information


Figures S1–S3.

Tables S1–S8.

## Data Availability

All new haplotype sequences of cpDNA and nrITS identified in this study have been deposited in GenBank under accession numbers OR916124–OR916134 and OR898905–OR898934, respectively. The DNA sequences of each individual used in this study have been stored in the Dryad data repository (https://datadryad.org/stash/share/DRaI6Ba5MLUgCgmLDzPrb_y8oudRyHUCoeVAW05BBmk).
