# Supplementary material for: Phylogeographic analysis reveals multiple origins of the desert shrub Reaumuria songarica in northern Xinjiang, involving homoploid and tetraploid hybrids
Source: Ecol Evol. 2024 Aug 29;14(9):e70199. doi: 10.1002/ece3.70199 (PMC11362504; doi:10.1002/ece3.70199)
Supplement: Supplementary file 1 — Figures S1–S3. [file ECE3-14-e70199-s001.docx]

**Supplementary information**

**Phylogeographic analysis reveals multiple origins of the desert shrub *Reaumuria songarica* in northern Xinjiang, involving homoploid and tetraploid hybrids**


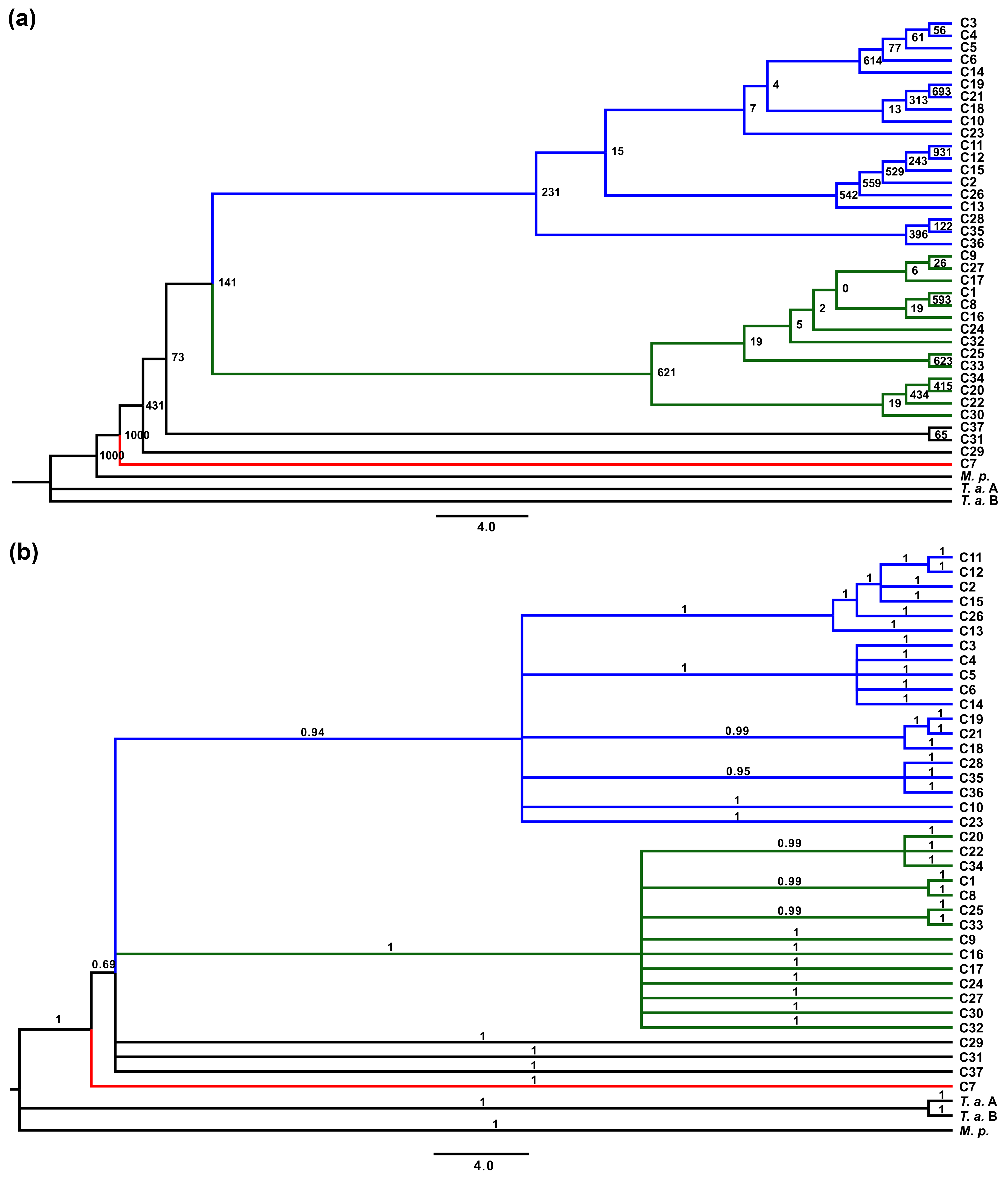


**Figure S1.** Phylogenetic trees of cpDNA haplotypes of *Reaumuria songarica*. Numbers above the branches indicate the bootstrap values calculated by maximum likelihood methods (a) and the posterior probabilities calculated by Bayesian inference (b), respectively. *M. p.*, *Myricaria pulcherrima*; *T. a*., *Tamarix amplexicaulis*.





**Figure S2.** Spatial genetic structure of *R. songarica* populations identified by cpDNA sequences. (a) The SAMOVA results of all populations. (b) The results of the GuD lineage.





**Figure S3.** Maternal gene flow between genetic groups of *R. songarica* estimated using MIGRATE-N. In each line chart, the direction of gene flow is from the first genetic group to the second. For example, GuD-TaD indicates gene flow from GuD to TaD. *M* = *m*/μ, where *m* represents the fraction of the new immigrants in the population per generation.
